# Ultra-High-Molecular-Weight Polyethylene in Hip and Knee Arthroplasties

**DOI:** 10.3390/ma16062140

**Published:** 2023-03-07

**Authors:** Masahiro Hasegawa, Shine Tone, Yohei Naito, Akihiro Sudo

**Affiliations:** Department of Orthopaedic Surgery, Mie University Graduate School of Medicine, Tsu 514-8507, Japan

**Keywords:** ultra-high-molecular-weight polyethylene, highly crosslinked polyethylene, hip, knee, arthroplasty, oxidation, vitamin E

## Abstract

Ultra-high-molecular-weight polyethylene (UHMWPE) wear and particle-induced osteolysis contribute to the failure of total hip arthroplasty (THA) and total knee arthroplasty (TKA). Highly crosslinked polyethylene (HXLPE) was developed in the late 1990s to reduce wear and has shown lower wear rates and loosening than conventional UHMWPE in THA. The irradiation dose for crosslinking is up to 100 kGy. However, during crosslinking, free radical formation induces oxidation. Using HXLPE in THA, the cumulative revision rate was determined to be significantly lower (6.2%) than that with conventional UHMWPE (11.7%) at a mean follow-up of 16 years, according to the Australian Orthopaedic Association National Joint Replacement Registry. However, HXLPE does not confer to TKA the same advantages it confers to THA. Several alternatives have been developed to prevent the release of free radicals and improve polymer mechanical properties, such as thermal treatment, phospholipid polymer 2-methacryloyloxyethyl phosphorylcholine grafting, remelting, and vitamin E addition. Among these options, vitamin E addition has reported good clinical results and wear resistance similar to that of HXLPE without vitamin E, as shown by short-term clinical studies of THA and TKA. This review aims to provide a comprehensive overview of the development and performance of UHMWPE in THA and TKA.

## 1. Introduction

Total joint arthroplasty, especially of the hip and knee, is a common and highly effective surgery [1]. Since its introduction in the 1960s, ultra-high-molecular-weight polyethylene (UHMWPE) has remained the most commonly used bearing material in total hip arthroplasty (THA) and total knee arthroplasty (TKA) [2]. The long-term failure rates of THA and TKA are concerning. UHMWPE wear is one of the most important post-surgical problems. Improvement in UHMWPE is essential for longevity after THA and TKA.

The manufacturing process of UHMWPE, component position, and patient factors can influence polyethylene wear. For example, crosslinking occurs during sterilization using gamma radiation. Since sterilization with conventional gamma radiation induces fatigue and higher rates of wear via air-initiated oxidation, it is currently performed in an inert environment [3]. Additionally, non-crosslinked polyethylene sterilized with ethylene oxide produces more wear than that sterilized with gamma radiation in ambient air [4,5].

In the late 1990s, it was shown that increasing the crosslinking improves the wear resistance of UHMWPE [6], with highly crosslinked polyethylene (HXLPE) showing lower wear rates than conventional UHMWPE. However, this has led to complications due to residual free radicals and compromised mechanical properties. To address these issues, several solutions have been recommended, such as antioxidant vitamin E addition [7,8]. This review aims to provide orthopedic surgeons and material engineers with a comprehensive overview of the development and performance of UHMWPE for improving longevity after THA and TKA. The novelty of this review consists in providing material improvements, pathological details, and clinical results in THA and TKA. The development of HXLPE has been one of the most useful innovations in THA. Using HXLPE, improvements have been observed in long-term results after THA. To further improve longevity, nanocomposite materials could be considered candidates for future research studies.

## 2. Conventional UHMWPE

Polyethylene is an ethylene polymer ((C_2_H_4_)_n_). UHMWPE consists of 200,000 ethylene repeat units, with up to 400,000 carbon atoms, and has crystalline lamellae, an amorphous region, and a third phase [9].

The molecular weight of medical-grade UHMWPE is between 3.5 and 6 million g/mol, with crystallinity ranging approximately between 50% and 55%. The American Society for Testing and Materials (ASTM) defines UHMWPE as having a molecular weight greater than 3.1 million g/mol. The International Standards Organization (ISO 11542) (ISO, 2001) specifies that UHMWPE is associated with a molecular weight of at least 1 million g/mol [10,11].

**Figure 1:**
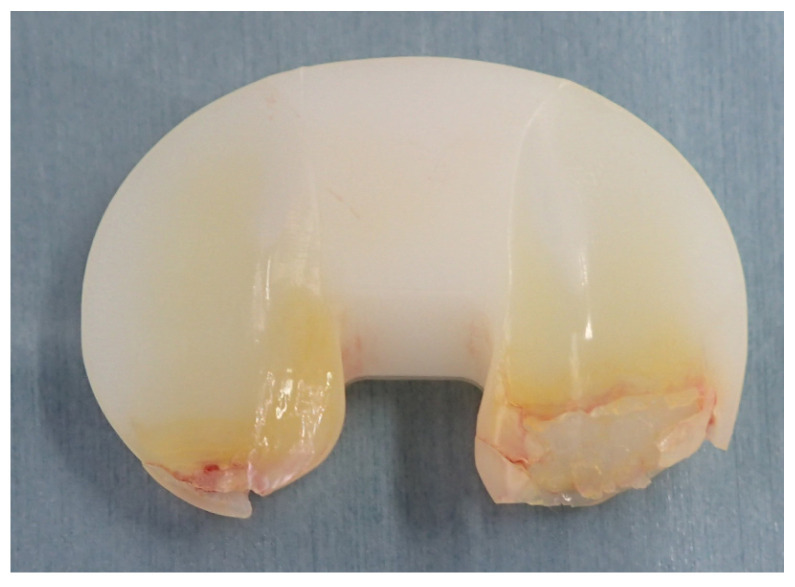
Retrieved conventional ultra-high-molecular-weight polyethylene after total knee arthroplasty showing severe wear and delamination.

Conventional UHMWPE previously employed gamma-radiation-in-air (gamma-air) sterilization at a dose of 25 kGy (2.5 Mrad). However, gamma radiation can break the C–C bonds of the polyethylene chain and create free radicals during crosslinking [2]. Sterilization with gamma-air initiates oxidation, leading to bearing fatigue and a high wear rate; thus, gamma irradiation sterilization in an inert environment (gamma-inert) was developed, in which oxygen-barrier packaging prevents the self-oxidation of the polymer. However, previously, when gamma-air- and gamma-inert-sterilized polyethylene was oxidized in vivo, in both cases, severe wear and failure occurred (Figure 1) [3]. Ethylene oxide gas and gas plasma sterilization have been introduced as alternatives to gamma-air sterilization in the 1970s and 1990s, respectively. While ethylene oxide gas is highly toxic, gas plasma does not leave any toxic residues [12]. Nevertheless, oxidation can still occur under both these conditions [13].

Electron-beam (e-beam) sterilization has also been explored as a potential method for the sterilization of UHMWPE. For example, the degradation of electron-beam-sterilized conventional UHMWPE tibial plates was evaluated. While the resistance to the oxidative degradation of e-beam sterilization was comparable to that of gamma-air sterilization, it was still inferior to that of gamma-inert sterilization [14]. Alternatively, to improve the wear resistance of UHMWPE, carbon-fiber-reinforced polyethylene (Poly Two; Zimmer, Warsaw, IN, USA) and UHMWPE with increased crystallinity (Hylamer; DePuy-DuPont Orthopedics joint venture, Newark, DE, USA) were introduced; however, their use was ultimately discontinued because of unacceptable rates of wear [15,16].

## 3. Polyethylene Wear, Osteolysis, and Loosening

**Figure 2:**
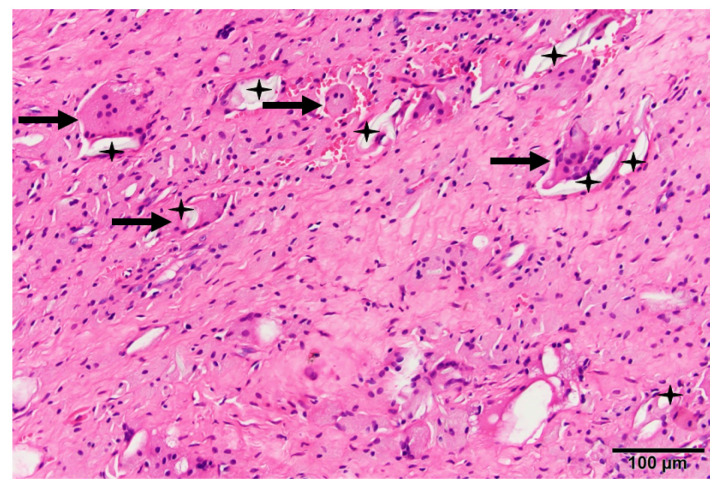
Histology of periprosthetic tissue with loosened metal on polyethylene total hip arthroplasty showing a number of multinucleated foreign-body giant cells (arrows), macrophages, and wear debris of polyethylene (stars). Hematoxylin-and-eosin staining.

Unlike metallic debris, fibrosis, fibrin exudation, and tissue necrosis are rarely found within the joint cavity along polyethylene wear particles and are accompanied by foreign-body giant cells and macrophages (Figure 2) [17]. The debris generated by polyethylene wear triggers a cascade of macrophage cytokines, including tumor necrosis factor (TNF)-α, interleukin (IL)-1β, and IL-6, causing osteoclastic bone resorption, which leads to osteolysis and aseptic loosening [18,19]. TNF-α is a critically potent inflammatory mediator of particle-induced bone resorption. Mice not expressing both p55 and p75 TNF receptors were protected from particle-induced osteolysis. Therefore, targeting TNF-α and/or its p55 receptor can prevent wear-particle-induced osteolysis [20,21].

Chemokines and their receptors are involved in the progression of periprosthetic osteolysis associated with aseptic loosening [22]. An increase in the gene expression of chemokines including CCL2, CCL3, CXCL8, CXCL9, and CXCL10 was demonstrated in periprosthetic tissues with aseptic loosening [23]. Current findings based on cell lines and animal models demonstrated several interactions of chemokine–chemokine receptors, such as CCR1–CCL3, CCR2–CCL2, CXCR2–CXCL2, and CXCR4–CXCL12, which have a crucial involvement in osteolysis. CCL2 is one of the most abundantly released chemokines, and it is an immediate, early stress-responsive factor that regulates systemic and local macrophage recruitment in chronic inflammation. CCL2 signals via C-C chemokine receptors 2 and 4 (CCR2/CCR4). Further, the CCR2–CCL2 axis has been suggested to play the most central role [23,24,25]. With the blocking of the interaction of chemokine–chemokine receptors, there was a substantial reduction in the osteolytic activity in a murine osteolysis model, which suggested that chemokine receptors play a crucial role in the progression of osteolysis.

Another pathway of osteoclast activation involves the stimulation of transcription factor nuclear factor kappa B (NF-κB) in osteoclast precursor cells. Receptor activator of NF-κB ligand (RANKL) is a ligand that is required for osteoclast generation. Further, receptor activator of NF-κB (RANK) is a receptor for RANKL, and osteoprotegerin (OPG) is a decoy receptor for RANKL [26]. Notably, RANKL stimulates RANK on the surface of osteoclast precursors. The intravenous administration of recombinant human OPG in mice reduced osteoclast activity and increased cancellous bone volume and density [27]. Gene therapy using a recombinant adeno-associated viral vector expressing OPG inhibited wear-debris-induced osteolysis in mice [28,29]. Denosumab is a human anti-RANKL neutralizing antibody that blocks the binding of RANKL to RANK, thereby inhibiting osteoclast activity and function. Denosumab is now clinically available for the treatment of osteoporosis and cancer-induced bone diseases [26]. A human trial for periprosthetic osteolysis is underway using denosumab. In a previous study, patients with known osteolysis in the proximity of an uncemented acetabular component ≥7 years after THA were randomized in a 1:1 ratio for subcutaneous injections of 60 mg denosumab or placebo for a total of six doses, with initiation on day 1 and, thereafter, every 6 months, with the last treatment being at 30 months [30]. No studies have reported its efficacy based on the therapeutic effect on humans.

The use of HXLPE liners reduced the incidence of osteolysis, loosening, and revision after THA over a follow-up period of up to 15 years [31]. However, this was not the case with TKA, as the incidence of the abovementioned events was similar between knees using HXLPE and conventional UHMWPE after TKA [32].

## 4. First-Generation HXLPE

To reduce polyethylene wear, “first generation” HXLPE was developed in the late 1990s (Table 1). The crosslinking of HXLPE linearly increased up to a radiation dose of 100 kGy, above which a plateau was attained [33]. With higher doses of radiation, the tensile and fracture toughness values became unacceptably low, while lower radiation doses resulted in better mechanical properties and lower wear resistance. It has been noted that the mechanical and fatigue strength of UHMWPE decreases after irradiation [10,34,35]. HXLPE is susceptible to fatigue crack as its inception stress intensity is reduced in comparison with conventional, unaged UHMWPE [35,36]. The limited ductility of HXLPE reduces the strain to failure and limits the polymer ability to accommodate plasticity at the crack tip. This is extremely important for fatigue crack propagation, since decreased plasticity at the crack tip enables more of the crack driving force to be utilized for crack progression rather than letting it be dissipated through the plastic work. With an increase in crosslink density, there is a concomitant decrease in propagation resistance, as indicated by the decreasing values of ΔK_incep_. Further, ΔK_incep_ decreased by 35% and 50% below non-crosslinked UHMWPE at 50 kGy and 100 kGy radiation dosages, respectively (Table 2) [36]. Much debate remains regarding the optimum dose of radiation, and implant manufacturers often choose either 50 or 100 kGy [37]. 

**Figure 3:**
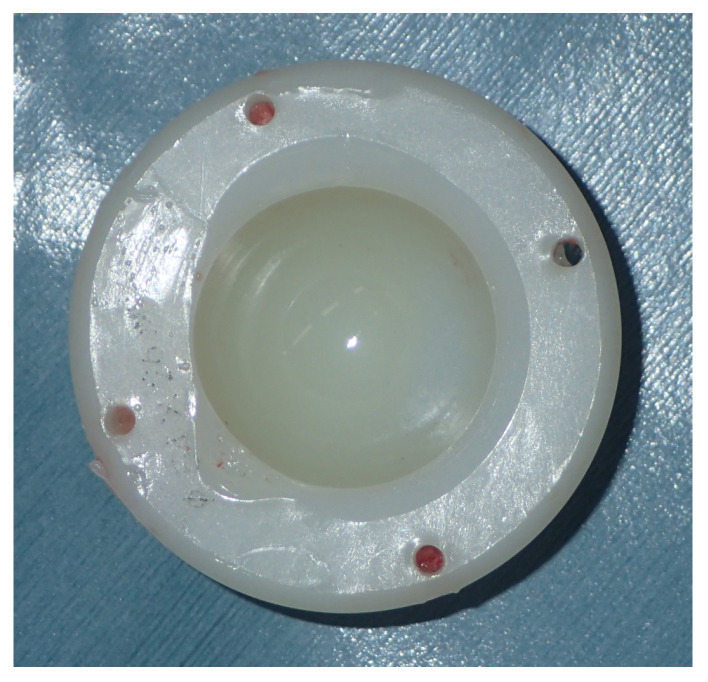
Retrieved annealed highly crosslinked polyethylene after total hip arthroplasty showing rim deformation due to impingement. The surface of the weight-bearing area was yellowish and considerably oxidized.

Thermal treatment of HXLPE, including annealing and remelting, was introduced to reduce the free radical formation and oxidation that occur during crosslinking. The melting point of HXLPE is approximately 140 °C, and free radicals are still present after annealing below this point (Figure 3). Annealing preserves the crystalline structure, while remelting, performed by heating UHMWPE above its melting point, eliminates free radicals; however, recrystallization is hindered after melting, thereby reducing the mechanical properties of UHMWPE [2,13,38]. Additionally, low levels of oxidation were detected in the retrieved remelted HXLPE. The mechanical properties of UHMWPE degrade when the degree of oxidation exceeds 1.0–1.5, resulting in the increase in the probability of delamination and cracking [39]. In middle-term samples, peaks in the oxidation index (OI) were observed on the subsurface (maximum OI = 4.5) and were induced by the combined effects of lipid absorption, mechanical stress, and ex vivo shelf-aging in air [40]. 

Larger femoral heads in THA have resulted in the use of thinner liners. Surgeons should consider the mechanical properties of thinner HXLPE, given that several cases of rim fractures in thin HXLPE liners have been reported [41,42,43]. Remelted HXLPE liners are recommended to be used at a thickness of at least 7 mm in weight bearing. At the rim, the minimum thickness was allowed to be 4.8 mm [43].

Additional lubrication was developed by grafting 2-methacryloyloxyethyl phosphorylcholine (MPC; Aquala, Kyocera, Kyoto, Japan) to the polymer. The MPC polymer has a side chain composed of phosphorylcholine, resembling the phospholipids of the biomembrane, and its surface yielded high-wettability and low-friction properties. Furthermore, the MPC polymer has been clinically used on the surfaces of intravascular stents, intravascular guidewires, soft contact lenses, and artificial lungs. To reduce UHMWPE wear and eliminate osteolysis, a novel UHMWPE liner with MPC grafted onto its surface (Aquala) has been developed [44,45,46]. For MPC grafting, polyethylene liners are placed in MPC solution after being coated with benzophenone as a photosensitizer, followed by photoinduced polymerization on the liner surface with an ultra-high-pressure mercury lamp [45]. The clinical and radiographic outcomes of MPC-grafted HXLPE liners 5 years after THA reported the mean steady-state wear rate to be very low (0.002 mm/y) [47]. 

Despite the favorable data collected with in vitro simulation and promising clinical reports [45,46,47,48], the MPC layer peeled off from the bearing surfaces of short-term-retrieved liners in several studies [49,50]. Additionally, MPC grafting also does not protect HXLPE against oxidation [49], and the extent of surface oxidation and the presence of different alkoxyl CO• radical species have been previously revealed [50].

## 5. Second-Generation HXLPE

The clinical introduction of thin acetabular liners with first-generation HXLPE raises the problem of the poor mechanical properties associated with them [51]. Second-generation HXLPE has been developed to improve the mechanical properties and reduce the generation of free radicals using methods other than gamma irradiation alone. Examples include sequential gamma irradiation followed by annealing (X3; Stryker, Mahwah, NJ, USA) [52,53], vitamin E diffusion or blending [2,7,8], and an alternative antioxidant blend (AOX; DePuy Synthes, Warsaw, IN, USA) [54]. Many manufacturers have developed second-generation HXLPE (Table 3), with most brands selling vitamin E-blended HXLPE.

A compression-molded GUR 1020 was used in X3 (Stryker) because of its higher ductility and impact strength than GUR 1050. The crosslinking of X3 was achieved in three cycles with a sequential irradiation-and-annealing process. Each cycle consisted of gamma irradiation at 30 kGy followed by annealing at 130 °C. The total radiation dose used was 90 kGy [52].

α-Tocopherol, a synthetic form of vitamin E and biological antioxidant, has been added to HXLPE to obtain oxidation resistance with improved fatigue strength by preventing post-irradiation melting [2,7,8]. Vitamin E can be added to HXLPE using two different procedures: before (blended vitamin E) or after crosslinking (diffused vitamin E). In the former, vitamin E is mixed with UHMWPE resin powder before consolidation and irradiation. The presence of vitamin E in UHMWPE during irradiation can protect the polymer from oxidation but decreases crosslink formation, thereby limiting the vitamin E concentration to ±0.3 wt%. In the latter, consolidated UHMWPE is infused with vitamin E after irradiation. Since vitamin E is not present during irradiation, the crosslinking efficiency of UHMWPE is not adversely affected. With this method, vitamin E concentration is limited to ±0.7 wt% [10,55].

An alternative antioxidant formulation for sterilization is the hindered phenol antioxidant pentaerythritol tetrakis (3-(3,5-di-tertbutyl-4-hydroxyphenyl) propionate) (COVERNOX, AOX; DePuy Synthes) [54]. 

## 6. Creep and Wear of UHMWPE

Radiographs show head penetration into the acetabular liner after THA using UHMWPE liner. Extensive studies were performed using radiographs after THA. However, the linear penetration measured using radiographs represents the cumulative contribution of creep and wear. Creep refers to a permanent deformation that occurs under loading and does not recover after load release. Wear involves both the delamination and progressive peel-off of surface flakes of UHMWPE, which results in the formation of highly reactive debris. It is desirable to distinguish creep and wear, thus preventing the misinterpretation of the tribological performance of UHMWPE liners [56]. Wear induces biological reactions such as osteolysis. However, creep does not induce adverse reactions, and it is important to differentiate creep from wear. Although wear has been reduced using HXLPE, creep has not been reduced [57,58]. 

When creep was measured using coordinate-measuring machines during loading without motion to 4 million cycles, the maximum creep occurred early in the loading cycle, with 90% of ultimate creep occurring before 2 million cycles in the liners with a 32 mm head and before 2.5 million cycles in the liners with a 28 mm head [57]. In clinical studies, the first one-year penetration has been considered to be creep. Steady-state wear has been defined as penetration after one year [58,59,60,61].

Using Raman spectroscopy, creep and wear can be quantitatively differentiated in retrievals, thus giving a correct estimate of the tribological performance of the studied implant. The two degradation mechanisms of creep and wear might have negative interactions, since the permanent deformation of the original surface of UHMWPE liner leads to femoral head migration and enhanced friction. Creep is not accompanied by irreversible mass loss from the material, unlike wear, but involves the packing and adjustment of UHMWPE molecules under compression. Once compressive (plastic) strain becomes locally measurable with Raman spectroscopy, in-depth Raman scanning can be used to evaluate thickness reductions due to creep [56]. Creep was measured in retrievals after THA using a calibrated relationship between full width at half maximum (FWHM) and strain measured with Raman spectroscopy [40,62]. In addition, Raman spectroscopy can measure the creep of tibial inserts retrieved after TKA [63]. However, the penetration of UHMWPE after TKA is hard to measure using radiographs because of the tilting of the tibial base plate relative to the plane of the radiograph.

## 7. Surface Damage of UHMWPE

**Figure 4:**
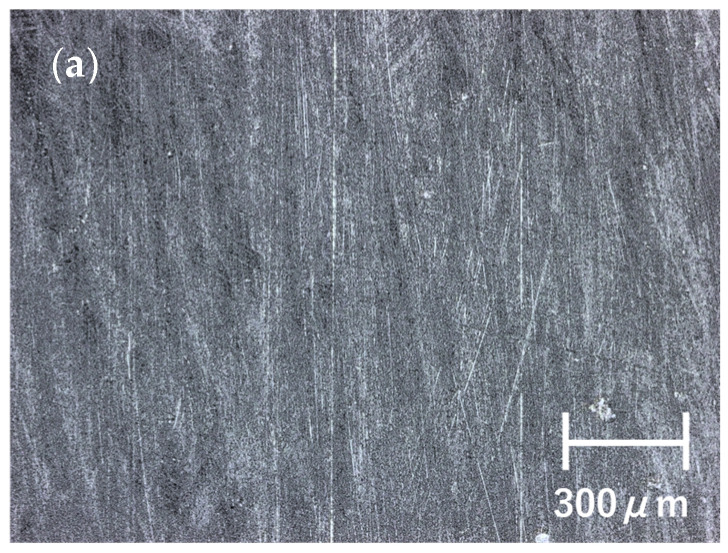

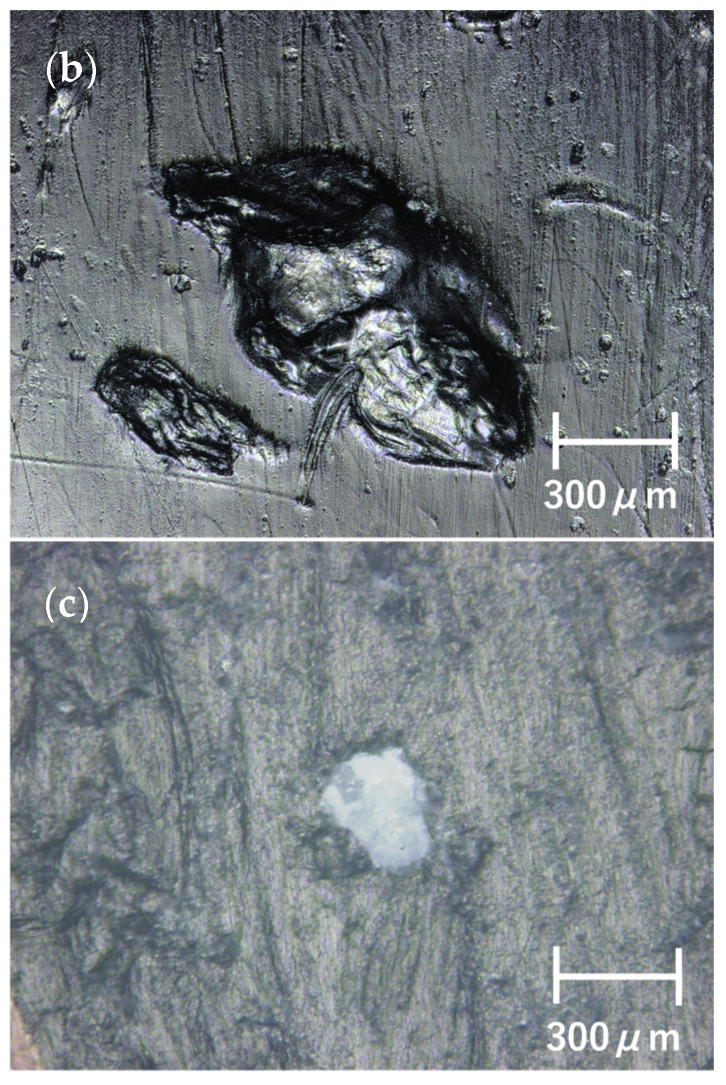
Optical micrographs of retrieved ultra-high-molecular-weight polyethylene. (**a**) Burnishing, (**b**) pitting, and (**c**) embedded debris.

Surface damage of retrieved UHMWPE is usually assessed using the Hood Score [64]. Seven modes of surface damage were reported: burnishing, scratching, pitting, abrasion, delamination, surface deformation, and embedded debris (Figure 4). Burnishing is characterized by the apparition of highly polished zones. Burnishing is characteristic of adhesive/abrasive wear, which produces micrometer-sized wear debris. This small debris could induce osteolysis. Scratching is identified as linear features on the surface, produced by protrusion on the opposing component surface or by third-body debris, and classified as a mode of abrasive wear. Pitting is characterized as surface defects of 2–3 mm in diameter and 1–2 mm in depth and is also referred to as cratering. Pitting is classified as a mode of fatigue wear with the disappearance of millimeter-sized wear debris from the surface. Pitting rarely provokes osteolysis because of large-size wear. Abrasion has a tufted or shredded appearance and is classified as a mode of abrasive wear. Delamination is characterized by surface from which a large sheet of UHMWPE has been removed. Delamination is a more severe damage mode than pitting. When the UHMWPE component is thin, delamination can result in catastrophic wear of UHMWPE. Surface deformation indicates permanent deformation on the articular surface. It is referred to as cold flow, creep, or plastic deformation without material removal. Embedded debris is debris entirely or partially pressed into the surface of UHMWPE and can scratch the opposing surface, resulting in further abrasive wear [65,66].

Backside wear should be paid attention to as a potentially clinically relevant source of wear debris. Backside wear is typically characterized as burnishing or scratching of UHMWPE. A smooth surface finish could be required for reducing backside wear, and a peripheral locking mechanism could contribute to backside wear [67].

When retrieved conventional acetabular liner and HXLPE liner after THA were compared, the articular surface damage modes were most commonly burnishing, pitting, and scratching, with no significant differences in damage modes between the two liners. Delamination was not found in any of the retrievals [68]. Retrieval studies were performed using conventional tibial inserts and HXLPE inserts after TKA, and most inserts (both conventional and HXLPE) exhibited some burnishing, pitting, scratching, and abrasion. No inserts exhibited delamination [69]. Considering the superior wear resistance of HXLPE, surface damage was an unexpected finding after THA and TKA. In a retrieval study after TKA, surface damage was compared between vitamin E-stabilized and first-generation HXLPEs, and the damage was similar [70]. When a retrieved conventional tibial insert from PFC and an antioxidant HXLPE insert from Attune (AOX; DePuy Synthes) after TKA were compared, there were no significant differences in surface damage [71]. However, Attune tibial inserts (AOX; DePuy Synthes) with fixed bearings showed significantly worse scores on the backside surface when compared with their PFC counterparts (conventional UHMWPE) [71]. The locking mechanism of UHMWPE could affect backside wear. The locking mechanism of PFC covers the entire peripheral UHMWPE and provides limited room for movement between UHMWPE and the metal tray, minimizing rotational micromotion. The locking mechanism of Attune has only three-point locking features that hold the UHMWPE tibial insert in place, leaving the lateral sides open [71].

## 8. Registry Data

Registries record, monitor, analyze, and report on performance outcomes in joint arthroplasty to ultimately improve patient outcomes.

### 8.1. Hip

In the Australian Orthopaedic Association National Joint Replacement Registry, the cumulative revision rate was significantly lower with HXLPE (6.2%) than with conventional polyethylene (11.7%) at a mean follow-up of 16 years [72]. In the National Joint Registry (NJR) for England, Wales, and Northern Ireland, the cumulative incidence rates of revision for aseptic loosening at 12 years were 0.52 and 0.54 per 100 THAs with HXLPE with total radiation dosages of ≥50 kGy to 100 kGy and ≥100 kGy, respectively. Additionally, this incidence was 1.95 per 100 THAs using no-radiation UHMWPE. In the same registry, UHMWPE with a total radiation dose of ≥50 kGy resulted in higher survival [73]. According to the New Zealand register, the annual revision rate per 100 implants was 0.54 with HXLPE versus 0.77 with conventional UHMWPE in ceramic femoral heads; in metal heads, the rates were 0.56 with HXLPE and 0.76 with conventional UHMWPE [5]. According to the Danish hip arthroplasty register, at a median 5-year follow-up, vitamin E-doped HXLPE had lower risk of revision for HXLPE-related endpoints than non-vitamin E HXLPE. However, higher risk of all-cause revision within 3 months has been reported with the former when compared with the latter. These revisions were primarily due to periprosthetic fractures and other causes unrelated to vitamin E-doped HXLPE [74]. According to the Finnish Arthroplasty Register, the 7-year survival of vitamin E-diffused HXLPE and non-vitamin E-diffused HXLPE with revision for any reason as the endpoint was comparable (94% and 93%, respectively). When the endpoint was revision because of aseptic loosening, the survival rate was 99% in both groups [75].

### 8.2. Knee

In the Australian Orthopaedic Association National Joint Replacement Registry, the 10-year cumulative revision rate with conventional UHMWPE was significantly higher than that with HXLPE (5.8% vs. 3.5%). The lower rate of revision was most evident in patients of <65 years of age [76]. In contrast, all-cause and aseptic revision rates were significantly lower with conventional UHMWPE than with HXLPE after a maximum follow-up duration of 12 years according to the NJR for England, Wales, and Northern Ireland [77]. In the American Joint Replacement Registry, compared with conventional UHMWPE, there were no differences in all-cause revision and aseptic revision with HXLPE, with a median follow-up of 3 years [78]. Furthermore, there were no differences in revision risk between HXLPE with or without an antioxidant and conventional UHMWPE [78].

## 9. Clinical Results with Second-Generation HXLPE

Long-term results with second-generation HXLPE were not provided in registry data.

The clinical performance of each second-generation HXLPE varies with respect to several aspects. In THA using sequentially irradiated and annealed HXLPE (X3; Stryker), the mean linear wear rate was 0.02 ± 0.03 mm/y with a mean follow-up of 10 years, and the all-cause survival rate was 92% [79]. Another study demonstrated that the mean linear wear rate was 0.085 mm, and the overall survival rate for all-cause revision was 95% at a mean follow-up of 13 years [80]. The long-term results were excellent, although in vivo oxidation has been reported [81,82].

Mid-term results with vitamin E-diffused HXLPE after THA have been reported in many studies, with contrasting results (Table 4) [83,84,85,86,87,88]. Several five-year randomized controlled trials demonstrated significantly lower wear for vitamin E liners compared with non-vitamin E HXLPE [83,85,87]. However, another study showed no differences between hips with and without diffused vitamin E after 5 years [88]. A seven-year randomized controlled trial showed that polyethylene wear did not differ between hips with and without vitamin E-diffused HXLPE [86]. Furthermore, patient-reported outcome measures did not differ between hips with and without vitamin E-diffused HXLPE [85,88]. A 5-year randomized controlled trial using vitamin E-blended HXLPE (Vitelene; Aesculap AG, Tuttlingen, Germany) showed no differences in wear rate compared with non-vitamin E HXLPE [89]. However, in another study, vitamin E-blended HXLPE (RM uncemented monoblock Pressfit Vitamys cup; Mathys, Bettlach, Switzerland) showed a lower wear rate (0.028 mm/year) than the UHMWPE cup (0.035 mm/year) after six years [90].

In TKA, studies reporting the performance of HXLPE with vitamin E and an alternative antioxidant after a 5 year follow-up are lacking. An analysis of the American Joint Replacement Registry with a median follow-up of 34 months demonstrated no differences in revision risk between HXLPE with or without an antioxidant and conventional UHMWPE [53]. In addition, the observed oxidation in retrieved vitamin E-diffused HXLPE was low, with a median OI of 0.09 for the articulating surface [91]. 

Without eliminating the mechanical properties of vitamin E-diffused HXLPE compared with remelted HXLPE, early fractures of vitamin E-diffused HXLPE liners after THA and TKA have been reported [92,93,94].

## 10. Effects of Femoral Head Material in THA and Femoral Component Material in TKA on UHMWPE Wear

Ceramic femoral heads were first introduced during the 1970s and are fabricated using alumina, zirconia, and other various composite powders that are compressed, sintered, and polished for use in THA. Pure alumina, zirconia, and zirconia-toughened alumina have been used as ceramic femoral heads. Ceramic femoral heads have harder and smoother surfaces than metal femoral heads and are more resistant to third-body damage; they have better wettability and decreased surface roughness, which can contribute to less polyethylene wear [95]. A randomized controlled trial with radiostereometric analysis demonstrated that ceramic heads (0.003 mm/year) showed no superiority in HXLPE wear over metal heads (0.007 mm/year) [96]. A systematic review and meta-analysis by Gosling et al. [97] demonstrated no differences in wear rates between ceramic and metal heads. Another systematic review and meta-analysis by Mertz et al. [95] compared the steady-state wear rates of ceramic and metal femoral heads with HXLPE liners. The mean wear rate was significantly higher with metal heads (0.063 mm/year) than with ceramic heads (0.047 mm/year). An alternative type of material that combines the strength of a metal with the surface properties of a ceramic is oxidized zirconium, which is a hard, highly wettable, monoclinic ceramic zirconium oxide surface on a zirconium metal implant (Oxinium; Smith & Nephew, Memphis, TN, USA). The surface oxide layer is not a coating but rather the surface zone of the metal alloy, conferring bearing properties to the ceramic head [98]. When comparing an oxidized zirconium femoral head and a metal femoral head using radiostereometric analysis, there were no statistically significant differences in steady-state wear rates between the oxidized zirconium (0.031 mm/year) and metal femoral heads (0.024 mm/year) [99]. In a systematic review and meta-analysis by Malahias et al. [98], oxidized zirconium heads did not lead to lower polyethylene wear rates than metal heads after THA (rate ratio: 0.836).

Using a ceramic femoral component in TKA, excellent clinical results with a minimum of 10-year follow-up period were reported [100]. The cumulative percent revision was 0.9% at 10 years [95]. A prospective comparative study of ceramic and metal femoral components in TKA at a more than five-year follow-up revealed that the outcomes of the ceramic femoral component demonstrated good clinical and radiological results, as well as survival comparable to that of the metal femoral component after TKA and failed to show superior results using a ceramic femoral component [101]. In a systematic review on ceramic femoral components including pure alumina, zirconia, and zirconia-toughened alumina in TKA, the ceramic components showed clinical results and survival rates similar to the metal components [102]. No comparative study of the UHMWPE wear of ceramic and metal femoral components was found. Oxidized zirconium was also used for the femoral component in TKA in an attempt to reduce UHMWPE wear and decrease aseptic loosening. Oxidized zirconium femoral components (Oxinium; Smith & Nephew) did not reduce revision rates for all causes compared to metal femoral components [103]. At 12 years, the cumulative percent revision rates were 4.8% with the metal prosthesis and 7.7% with the oxidized zirconium prosthesis [103].

## 11. Microorganism Adhesion on UHMWPE

Microorganism adhesion on UHMWPE has been identified as the first step in biomaterial-associated infection pathogenesis after THA and TKA. Although UHMWPE is used after several sterilization processes and microorganism adhesion on biomaterial surfaces depends on the physicochemical interactions between substratum and microorganism as well as the physical properties of the biomaterial surface (roughness, coating, surface energy, electrostatic charge, and hydrophobicity), *Staphylococcus epidermidis*, *Staphylococcus aureus*, *Escherichia coli*, and *Candida albicans* showed lower adhesion on HXLPE than on conventional UHMWPE in vitro [104]. However, clinical studies demonstrated no differences in infection between HXLPE and conventional UHMWPE [105]. Vitamin E may have the potential to reduce bacterial adhesion. HXLPE with vitamin E could have the potential to reduce the adhesive ability of *Staphylococcus epidermidis*, *Staphylococcus aureus*, and *Escherichia coli* in vitro [106,107]. However, the clinical relevance is questionable [56].

## 12. Future Direction of UHMWPE

UHMWPE belongs to an emerging class of high-performance, specialty polymers that has witnessed a phenomenal growth in research, development, and commercialization. However, the production of UHMWPE encounters many technological and scientific challenges, including designing appropriate metal catalysts, identifying suitable activators, identifying suitable reaction conditions for polymerization, balancing the electronic and steric properties of the metal center to minimize transfer and termination reactions, and the ability to produce UHMWPE in a disentangled state for easier processing [108].

Polymer nanocomposites are being investigated as alternatives to UHMWPE in joint arthroplasties [109]. High-density polyethylene (HDPE) has good mechanical properties, but the wear resistance is poor. Sahu et al. [110] evaluated the effect of contact pressure on the wear performance of HDPE reinforced with multidimensional carbon-based nanofillers. A direct relationship was drawn between contact pressure and the wear volume of composites and hybrids. The smallest contact pressure and best wear performance were noted with graphite nanoplatelet/nanodiamond composites, followed by nanodiamond nanocomposites. The cumulative effect of the presence of multi-walled carbon nanotubes and irradiation effectively increased the wear resistance of UHMWPE [111]. An emphasis on reinforcing UHMWPE with 1D nanofillers such as nanodiamonds has a special significance due to the extraordinary hardness, modulus of elasticity, and superior wear properties of this material [112]. The existence of nanodiamonds improved the surface properties and mechanical properties of UHMWPE [112]. The addition of graphene oxide nanoparticles to UHMWPE demonstrated a reduction in the wear of UHMWPE. UHMWPE/graphene oxide nanocomposites did not affect the inflammatory response to wear particles [109]. A new tribotechnical material, UHMWPE/CaSiO_3_ nanocomposites, was developed, and its high wear resistance was demonstrated [113].

## 13. Conclusions

UHMWPE is the most commonly used bearing material in THA and TKA. However, UHMWPE wear is one of the most important post-surgical problems. Improvement in UHMWPE is crucial for longevity after THA and TKA. The most important technology for reducing wear is HXLPE. In THA, first-generation HXLPE has contributed to reducing wear, osteolysis, and loosening compared with conventional UHMWPE. However, HXLPE does not confer to TKA the same advantages it confers to THA, as demonstrated by mid-term results. Future studies of HXLPE in TKA are needed to improve longevity. Nanocomposite materials could be candidates for future studies [109,110,111,112,113]. Second-generation HXLPE has been developed to improve the mechanical properties and reduce the generation of free radicals. Short-term clinical studies of THA and TKA using second-generation HXLPE reported good clinical results and low wear rates. Further studies on long-term HXLPE survival are needed. The clinical outcome of the novel materials is still largely unexplored.

## Figures and Tables

**Table 1:** First-generation highly crosslinked polyethylene.

Type	Brand (Manufacturer)	Irradiation, Dose (kGy)	Sterilization	Total Irradiation Dose (kGy)
Annealed	Crossfire (Stryker)	Gamma, 75	Gamma in nitrogen, 30 kGy	105
Annealed	ArCom XL (Zimmer Biomet)	Gamma, 50	Gas plasma	50
Annealed	Aeonian (Kyocera)	Gamma, 35	Gamma in nitrogen, 25 kGy	60
Annealed	Excellink (Kyocera)	Gamma, 50	Gamma in nitrogen, 25 kGy	75
Annealed	Aquala (Kyocera)	Gamma, 50	Gamma in nitrogen, 25 kGy	75
Remelted	Longevity (Zimmer Biomet)	Electron beam, 100	Gas plasma	100
Remelted	Prolong (Zimmer Biomet)	Electron beam, 65	Gas plasma or ethylene oxide	65
Remelted	Durasul (Zimmer Biomet)	Electron beam, 95	Ethylene oxide	95
Remelted	Marathon (DePuy Synthes)	Gamma, 50	Gas plasma	50
Remelted	XLPE (Smith & Nephew)	Gamma, 100	Ethylene oxide	100

**Table 2:** Mechanical properties and fatigue strength of ultra-high-molecular-weight polyethylene [36]. Reproduced with permission from Baker D.A. et al, Journal of Biomedical Materials Research Part A; published by John Wiley and Sons, 2003.

Radiation Dose (kGy)	Crystallinity (%)	Elastic Modulus (Mpa)	Yield Strength (Mpa)	True Stress at Break (Mpa)	ΔK_incep_ (MPa√m)
0	50.1	495	20.2	315.5	1.41
50	45.6	412	19.9	237.6	0.91
100	46.3	386	18.9	185.7	0.69

**Table 3:** Second-generation highly cross linked polyethylene.

Type	Brand (Manufacturer)	Irradiation, Dose (kGy)	Sterilization	Total Irradiation Dose (kGy)
Sequentially annealed	X3 (Stryker)	Gamma, 30 in 3 steps	Gas plasma	90
Vitamin E-diffused	E1 (Zimmer Biomet)	Gamma, 100	Gamma, 30 kGy	130
Vitamin E-blended	Vivacit-E (Zimmer Biomet)	Electron beam, not available	Ethylene oxide	Not available
Vitamin E-blended	Vitelene (Aesculap)	Electron beam, 80	Ethylene oxide	80
Vitamin E-blended	Vitamys (Mathys)	Gamma, 100	Gas plasma	100
Vitamin E-blended	ECiMa (Corin)	Gamma, 120	Ethylene oxide	120
Vitamin E-blended	Blend-E XL (Nakashima)	Electron beam, 300	Ethylene oxide	300
Vitamin E-blended	Aquala VE (Kyocera)	Gamma, 100	Gamma, 25 kGy	125
COVERNOX antioxidant-blended	AOX (DePuy Synthes)	Gamma, 85	Gamma, 30 kGy	115

**Table 4:** Comparison of polyethylene wear with vitamin E liner and non-vitamin E liner.

**Study**		Shareghi et al. [83]	Nebergall et al. [84]	Thoen et al. [87]
**Follow-up**	(years)	5	5	5
**Design**		RCT	RCT	RCT
**Material**	Vitamin E	E1	E1	E1
	Non-vitamin E	ArComXL	ArComXL	Marathon
**Wear (mm)**	Vitamin E	0.13	−0.05	0.17
	Non-vitamin E	0.2	0.07	0.2
Wear rate (mm/year) *	Vitamin E	0.02		
	Non-vitamin E	0.04		
**Results**		Significantly lower wear of vitamin E liner	Significantly lower wear of vitamin E liner	Significantly lower wear of vitamin E liner
**Study**		Kjærgaard et al. [88]	Galea et al. [85]	Galea et al. [86]
**Follow-up**	(years)	5	5	7
**Design**		RCT	Prospective study	RCT
**Material**	Vitamin E	E1	E1	E1
	Non-vitamin E	ArComXL	ArComXL	ArComXL
**Wear (mm)**	Vitamin E	0.006	0.06	−0.07
	Non-vitamin E	0.09	0.13	0
Wear rate (mm/year) *	Vitamin E	−0.006		
	Non-vitamin E	0.005		
**Results**		Not significant	Significantly lower wear of vitamin E liner	Not significant

* Two to five years. RCT: randomized controlled trial.

## Data Availability

Not applicable.
